# Structural and biological characterization of pAC65, a macrocyclic peptide that blocks PD-L1 with equivalent potency to the FDA-approved antibodies

**DOI:** 10.1186/s12943-023-01853-4

**Published:** 2023-09-07

**Authors:** Ismael Rodriguez, Justyna Kocik-Krol, Lukasz Skalniak, Bogdan Musielak, Aneta Wisniewska, Agnieszka Ciesiołkiewicz, Łukasz Berlicki, Jacek Plewka, Przemyslaw Grudnik, Malgorzata Stec, Maciej Siedlar, Tad A. Holak, Katarzyna Magiera-Mularz

**Affiliations:** 1 Department of Organic Chemistry, Faculty of Chemistry, Jagiellonian University, Gronostajowa 2, Krakow, 30-387 Poland; 2 Department of Bioorganic Chemistry, Faculty of Chemistry, Wrocław University of Science and Technology, Wybrzeże Wyspiańskiego 27, Wrocław, 50-370 Poland; 3 Malopolska Centre of Biotechnology, Jagiellonian University, Gronostajowa 7a, Krakow, 30-387 Poland; 4 Department of Clinical Immunology, Institute of Pediatrics, Jagiellonian University Medical College, Wielicka 265, Krakow, 30-663 Poland

**Keywords:** PD-1, Programmed death 1, PD-L1, Programmed death ligand 1, CD80, Immune checkpoint blockade, Cancer immunotherapy

## Abstract

Recent advances in immuno-oncology have opened up new and impressive treatment options for cancer. Notwithstanding, overcoming the limitations of the current FDA-approved therapies with monoclonal antibodies (mAbs) that block the PD-1/PD-L1 pathway continues to lead to the testing of multiple approaches and optimizations. Recently, a series of macrocyclic peptides have been developed that exhibit binding strengths to PD-L1 ranging from sub-micromolar to micromolar. In this study, we present the most potent non-antibody-based PD-1/PD-L1 interaction inhibitor reported to date. The structural and biological characterization of this macrocyclic PD-L1 targeting peptide provides the rationale for inhibition of both PD-1/PD-L1 and CD80/PD-L1 complexes. The IC_50_ and EC_50_ values obtained in PD-L1 binding assays indicate that the pAC65 peptide has potency equivalent to the current FDA-approved mAbs and may have similar activity to the BMS986189 peptide, which entered the clinical trial and has favorable safety and pharmacokinetic data. The data presented here delineate the generation of similar peptides with improved biological activities and applications not only in the field of cancer immunotherapy but also in other disorders related to the immune system.

**Supplementary Information:**

The online version contains supplementary material available at 10.1186/s12943-023-01853-4.

## Background

Antibodies directed at immune checkpoints have significantly changed the field of immuno-oncology and oncology in general [1]. A key target in this area is a protein-protein interaction (PPI) between programmed cell death 1 (PD-1) and its ligand, PD-L1. Functionally, PD-1 is an immune checkpoint molecule located mainly on T cells. The PD-1/PD-L1 axis is hijacked by some viruses during the infection, as well as by cancer cells to suppress immune surveillance. In particular, PD-L1 expressed on cancer cells inhibits the killing of tumor cells by T cells. Blocking either PD-1 or PD-L1 restores T-cell function and allows T cells to kill cancer cells [1, 2].

Besides PD-L1 binding to PD-1, previous studies have shown another ligand of PD-L1, CD80, which upon binding in a *Cis* mode provides complex cross-relation between PD-1, CTLA-4, and CD28 pathways [3]. Although this immune checkpoint cross-talking has become a hot topic of research, the immunological events caused by the blockade or formation of the PD-L1/CD80 complex are still under investigation.

Immune checkpoint blockade (ICB) with antibodies has shown impressive clinical results in the treatment of several types of tumors providing durable disease regression and even cure for a subgroup of cancer patients [1]. Therapeutic antibodies, however, exhibit several disadvantages such as poor tissue penetration, lacking oral bioavailability, potential immunogenicity, and accompanied immune-related adverse events (irAEs) [2]. In addition, the current ICB leads to a tumor response only in a fraction of cases and tumor types. Therefore, a search for non-biologics, including small molecules, peptides, cyclo-peptides, and macrocycles is ongoing [4–7].

In this study, we characterize the biological activity of a macrocyclic peptide-based immune checkpoint inhibitor named pAC65. This macrocyclic peptide was first listed in the Bristol Myers Squibb patent in 2016 [8] but no experimental evidence of the postulated inhibition of both the PD-L1/PD-1 and PD-L1/CD80 immune checkpoints or structural details for its interaction with PD-L1 was provided, till now. We recently provided a structural rationale for the activity of macrocycles containing 15, 14, and 13 residues with sub-micromolar to micromolar potency [5, 6]. Here, we demonstrate the structural and biological characterization of the first highly potent macrocyclic peptide with activity in the sub-nanomolar range displaying a potency comparable to clinically approved PD-L1 antibodies.

## Results

### pAC65 dissociates PD-L1/PD-1 and PD-L1/CD80 complexes in NMR assays

**Fig. 1:**
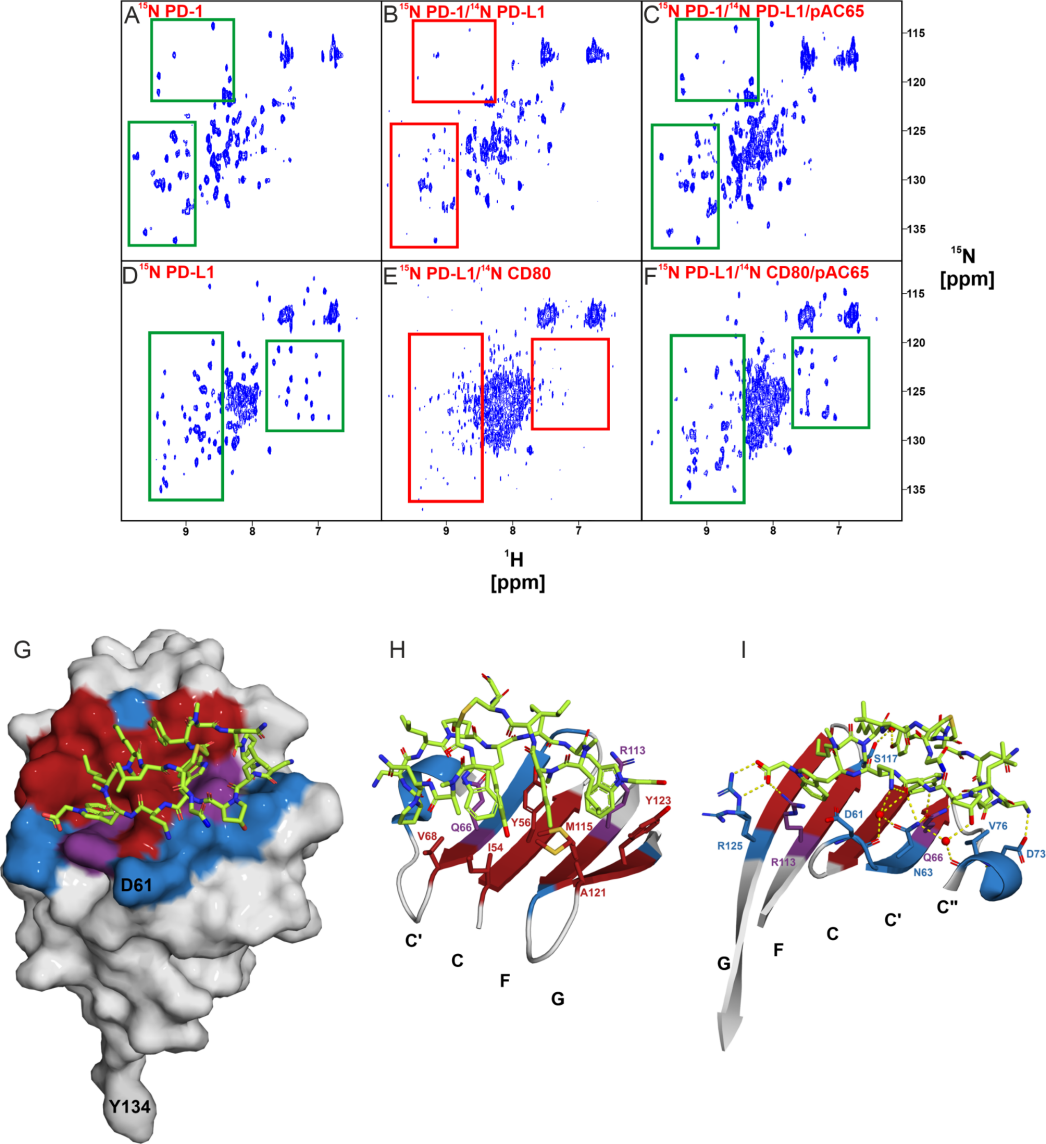
The pAC65 peptide binds to PD-L1 and disrupts the PD-L1/PD-1 and PD-L1/CD80 complexes. **A-F** 2D (^1^H-^15^N SOFAST-HMQC) AIDA experiments. The spectra of apo-PD-1, characteristic signals boxed in green (**A**), a complex of PD-1 and PD-L1, characteristic signals disappeared – boxed in red (**B**), the sample after the addition of peptide pAC65 to the PD-1/PD-L1 complex in the molar ratio 1:1 (**C**), apo-PD-L1 (**D**), a complex of PD-L1 and CD80 (**E**), the sample after the addition of peptide pAC65 to the complex PD-L1/CD80 in the molar ratio 1:1 (**F**). The characteristic signals in the spectra of apo proteins are boxed in green ((**A**) and (**D**)); signals disappeared upon complexes formation, boxed in red ((**B**) and (**E**)); restored peaks after the addition of pAC65 to the protein complexes are boxed in green ((**C**) and (**F**)) **G-I** Crystal X-ray structure of the PD-L1/pAC65 complex (PDB: 8ALX). **G** Overall view of the PD-L1/pAC65 binding interface, hydrophobic interactions are shown in red, hydrophilic in blue, and residues that provide both types of interactions are colored in violet. **H** Detailed hydrophobic interactions of pAC65 at the binding interface. Color-coded as in panel G. **I** Detailed polar interactions of pAC65 at the binding interface. Color-coded as in panel G

We used an NMR method to assess the ability of the pAC65 peptide to interact with PD-L1. Titration of the ^15^N-labeled PD-L1 with increasing amounts of the pAC65 peptide resulted in perturbations of the proton chemical shifts (^1^H NMR) and ^1^H-^15^N cross-correlation peaks, which were monitored using the SOFAST-HMQC. Significant changes in both ^1^H and 2D NMR spectra clearly indicated strong binding of the pAC65 peptide to the PD-L1 protein (Additional files 2 and 3: Fig. S1 and S2). To estimate the K_D_ value of the interaction of the pAC65 peptide with PD-L1, we used AIDA-NMR (Antagonist Induced Dissociation Assay-NMR) experiments [9]. To perform the first 2D AIDA experiment, the ^15^N labeled PD-1 was used as the reporter protein and titrated with unlabeled PD-L1 until the PD-1/PD-L1 complex was formed, as indicated by the broadening of NMR resonances and the disappearance of the signals in the ^1^H-^15^N SOFAST-HMQC spectra (characteristic peaks are highlighted in the boxes). Titration of the complex with pAC65 resulted in signal recovery indicating dissociation of the PD-1/PD-L1 complex upon peptide binding (Fig. 1A C). A similar result was obtained when the titration of the PD-L1/PD-1 complex with pAC65 was monitored by 1D AIDA NMR (Additional file 4: Fig. S3A). An additional binding study using 1D NMR was performed to assess the interaction between pAC65 and murine PD-L1. However, pAC65 does not exhibit any discernible binding affinity for murine PD-L1, evidenced by the lack of observable changes in the NMR spectral patterns, as shown in Figure S3B. The second 2D AIDA experiment was performed analogously using ^15^N-labelled PD-L1 as a reporter protein and an unlabelled CD80 protein. As before, addition of the pAC65 peptide to the preformed PD-L1/CD80 complex resulted in signal recovery, as monitored by both 2D AIDA and 1D AIDA NMR, indicating dissociation of the complex upon peptide binding (Fig. 1D F and Additional file 5: Fig. S4). Overall, titration of the PD-1/PD-L1 and PD-L1/CD80 complexes with pAC65 resulted in complete dissociation of the complexes (Additional files 4 and 5: Fig. S3 and S4). This indicates that pAC65 can inhibit both the PD-L1/PD-1 and PD-L1/CD80 complexes in vitro, while the estimated K_D_ value of the interaction of pAC65 with PD-L1 is in the nanomolar range (for comparison K_D_ of the PD-1/PD-L1 interaction ∼ 8 µM and K_D_ of the PD-L1/CD80 interaction ∼1.7 µM) [10].

The ability of the peptide pAC65 to dissociate the preformed PD-1/PD-L1 complex was additionally assessed using a commercially available Homogenous Time-Resolved Fluorescence (HTRF) interaction assay (Cisbio, France). The half-maximal inhibitory concentration (IC_50_) was derived by fitting the experimental data with the Hill’s model, yielding the value of 1.80 ± 0.16 nM with a good data-to-fit correlation (Additional file 6: Fig. S5).

### Structural basis of PD-L1 interaction with peptide pAC65

High-quality crystals were obtained for pAC65 in complex with PD-L1 that allowed solving the structure at the 1.1 Å resolution (Additional file 7: Table S1). The asymmetric unit of the pAC65/PD-L1 complex contains one molecule of PD-L1 and one molecule of the pAC65 peptide. The interaction between pAC65 and PD-L1 occurs on the plane of the PD-L1 β-sheet composed of strands G, F, C, and C’, as indicated by the canonical Ig designations depicted in Additional file 8: Fig. S6. The peptide ring assumes an ellipse-like shape with a bulge located above the central region and binds parallelly to the plane of the β-sheet. The backbone of the macrocyclic ring extends itself from the _65_Ala-NH_2_5 to _65_TrpNAc10 while the bulge is formed by _65_Tyr1, _65_NMeNle12, _65_Leu13, and _65_Scc14 (the subscript 65 indicates the macrocyclic peptide pAC65).

The macrocyclic ring is stabilized by various intramolecular interactions. Polar forces are important in maintaining the shape of the peptide and provide rigidity to the entire structure. The backbone forms three subsequent β-turns between _65_Asn3 carbonyl and _65_Leu6 amine, _65_Dab9 carbonyl and _65_Leu13 amine, and _65_Scc14 carbonyl and _65_Asn3 amine. _65_Leu13 carbonyl and _65_Dab9 amine form further hydrogen bonds which stabilize the central part of the ring whereas adjacent _65_Hyp7 carbonyl contributes a hydrogen bond to _65_Gly-NH_2_15 amine. The bulge located over the central region of the peptide ring is stabilized by polar contacts between _65_Tyr1 amine and _65_Leu13 carbonyl. The polar contacts are supplemented by weak hydrophobic interactions. The sidechain of _65_Tyr1 provides a T-shaped π stacking interaction with the indole moiety of _65_Trp8 while _65_NMeAla2 forms alkyl-π interaction with the sidechain of _65_Tyr1. The peptide ring contains one *cis*-peptide bond formed between _65_Tyr1 and _65_NMeAla2. The presence of this *cis* bond may be due an N-methylated amino acid (_65_NMeAla2) in the cyclic peptide sequence.

Most of the polar residues of the main chain are solvent-exposed while those non-polar ones are facing the PD-L1 interaction surface. The observed binding mode is similar to the other macrocycle/PD-L1 complexes previously reported by us [5, 6]. The interaction surface of the pAC65 peptide coincides with the binding sites of PD-1 and ALPN-202 (engineered CD80 vIgD) on the PD-L1 surface, providing a rationale for the inhibition of both PD-1/PD-L1 and CD80/PD-L1 pathways (Additional file 9: Fig. S7) [11]. The binding interface of the peptide includes hydrophobic interactions located at the center of the interaction area strengthened by polar contacts at the rim of the binding site (Fig. 1G and I and Additional file 10: Fig. S8C). The hydrophobic interlinkage is determined by the aromatic and non-aromatic moieties of _65_Pro4, _65_Trp8, _65_TrpNAc10, _65_NMeNle11, and _65_NMeNle12. Two indole moieties of _65_Trp8 and _65_TrpNAc10 occupy major hydrophobic clefts at the surface of PD-L1 and create the T-shaped stacking interactions with Tyr56 and Tyr123, respectively. The stacking interactions are supplemented with several alkyl-π interactions with Gln66, Arg113, and Met115. Although _65_TrpNAc10 binds within a prominently hydrophobic cleft, the acetic acid moiety of _65_TrpNAc10 extends the cleft towards Arg125 and Arg113 providing crucial salt bridges with the side chains of these amino acids (Fig. 1I). Adjacent to the cleft occupied by _65_TrpNAc10 two norleucine side chains of _65_NMeNle11 and _65_NMeNle12 provide non-polar interactions with Met115, Ala121, and Tyr123. The side chain of _65_Pro4 forms a weak hydrophobic interaction with the side chain of Val68. The aromatic ring of _65_Tyr1 provides hydrophobic interaction with Ile54 while its hydroxyl group contributes to water molecule-mediated hydrogen bond formation with the side chain of Ser117 which further supports binding.

Various polar interactions can be seen dispersed throughout the macrocyclic ring, besides those previously mentioned. The side chain amine of _65_Ala-NH_2_5 contributes a direct hydrogen bond with the side chain oxygen of Asp73. Two water-mediated hydrogen bonds are formed between the side chain amine of _65_Dab9 and the backbone oxygen of Asp61 and the side chain amine of _65_Dab9 and the side chain of Asn63. Further side chain amine of Asn63 contributes a direct hydrogen bond to backbone oxygen of _65_Trp8. The side chain of Gln66 participates in two hydrogen bonds with backbone amide of _65_Trp8 and backbone carbonyl of _65_Leu6. Moreover, the overall structure of the ring is stabilized by several other water-mediated interactions.

### In vitro bioactivity of the pAC65 peptide

**Fig. 2:**
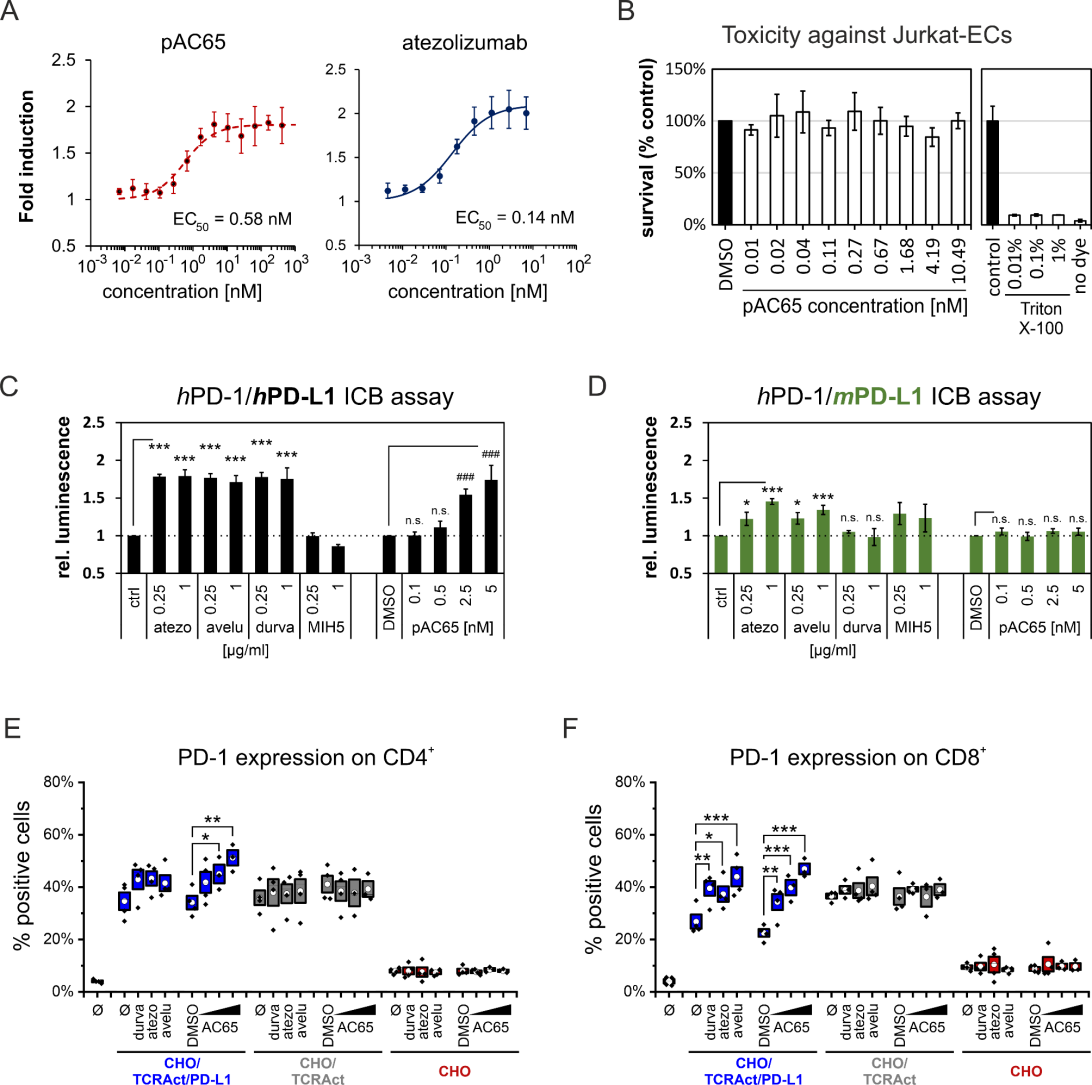
The pAC65 peptide restore the activation of Jurkat-ECs cell line and primary human T cells. **A** Dose-dependent reactivation of Jurkat-ECs with *h*PD-L1-blocking agents: pAC65 (left panel) and atezolizumab (right panel) in the ICB assay. Graphs show fold luminescence induction relative to either untreated (for atezolizumab) or DMSO-treated (for pAC65) cells. Data points represent mean ± SD values from 4–6 independent experiments. **B** The long-term (48 h) cytotoxicity of the peptide pAC65 towards Jurkat-ECs. The graph shows Jurkat-ECs survival relative to DMSO-treated control cells. Triton X-100-treated cells and cells without the addition of a Redox Dye were used as baseline controls. **C** The blockade of the human PD-L1 (*h*PD-L1). **D** The blockade of mouse PD-L1 (*m*PD-L1) in the ICB assay. In the assay, three antibodies (atezo. – atezolizumab, ave. – avelumab, durva. – durvalumab) and the peptide pAC65 were used. Graphs show fold luminescence induction relative to either untreated (for antibodies) or DMSO-treated (for pAC65) cells The expression of PD-1 on either CD4^+^ (**E**) or CD8^+^ (**F**) cells was determined by flow cytometry. PBMCs from healthy donors were exposed to either CHO/TCRAct/PD-L1, CHO/TCRAct, or CHO cells for two days in the presence of durvalumab (durva), atezolizumab (atezo), avelumab (avelu), or increasing concentrations of the peptide pAC65 (the concentrations: 2.5 nM, 25 nM, or 250 nM). ø indicates untreated cells. The graphs show fractions of positive cells and represent cumulative data from 3–4 donors. Posthoc test *, p < 0.05, **, p < 0.01, ***, p < 0.001, or DMSO-treated cells: ###, p < 0.001

To evaluate the bioactivity of the pAC65 peptide, cell-based immune checkpoint blockade assays, T-cell activation (TCA) assay, and a viability assay were performed. In the first, a classical ICB setup, the reporter Jurkat Effector cells (Jurkat-ECs) are co-cultured with the antigen-presenting cell surrogate CHO/TCRAct/PD-L1 (aAPCs) cells in the presence of a tested drug. Overexpression of a TCR-Activator molecule (TCRAct) assures the activation of Jurkat-ECs, while the inhibitory PD-1/PD-L1 checkpoint is provided by overexpression of human PD-L1 on CHO/TCRAct/PD-L1 cells and human PD-1 on Jutkat-ECs. In the assay, the pAC65 peptide dose-dependently increased the activation of Jurkat-ECs with the EC_50_ value of 0.58 nM, indicating a clear potential for PD-L1 blockade in a cellular context (Fig. 2A). A similar effect was observed for a therapeutic antibody atezolizumab, which restored the activation of Jurkat-ECs with the EC_50_ value of 0.14 nM (Fig. 2A). The pAC65 peptide did not present any toxicity toward the Jurkat-ECs in the concentration range used in the ICB assay (Fig. 2B), as tested in the cell viability assay (Fig. 2B). Lack of toxicity was previously observed also for another representative macrocyclic peptide PD-L1 blocker [6]. Moreover, our previous studies indicated that the molecules targeting human PD-L1 fail to target the mouse PD-L1 protein. To verify the blockade of the mouse PD-L1 (*m*PD-L1), the pAC65 peptide was tested in a modified ICB assay, in which the CHO/TCRAct/PD-L1 cells were substituted with B16-F10/TCRAct cells (*m*aAPCs), which express high levels of endogenous mouse PD-L1 upon the treatment with IFN-γ [12]. The peptide was tested along with three therapeutic antibodies, atezolizumab, avelumab, and durvalumab. As reported before [12], atezolizumab but not durvalumab was able to block the *h*PD-1/*m*PD-L1 immune checkpoint (Fig. 2D), while both antibodies efficiently blocked the *h*PD-1/*h*PD-L1 immune checkpoint (Fig. 2C). Avelumab reflected the activity of atezolizumab, in that it restored the activation of Jurkat-ECs blocked with either the *h*PD-L1-expressing or *m*PD-L1 expressing cells (Fig. 2C and D). The peptide pAC65 dose-dependently blocked the *h*PD-1/*h*PD-L1 in a classical ICB assay (Fig. 2C) but failed to block the *h*PD-1/*m*PD-L1 checkpoint (Fig. 2D) indicating a similar specificity towards the *h*PD-L1 protein, as observed before for a macrocyclic peptide p57 and small molecules BMS-1001 and BMS-1166 [12].

Next, the ability of the peptide pAC65 to restore the activation of primary human T cells was evaluated in the T-cell activation assay. In the assay, human primary peripheral blood mononuclear cells (PBMCs) are contacted with either CHO/TCRAct/PD-L1, CHO/TCRAct, or CHO cells alone or in the presence of PD-1/PD-L1-blocking molecules. The activation of CD4 + and CD8 + T cells is analyzed with flow cytometry. In the experiment, four T-cell activation markers were monitored, namely CD69, CD25, HLA-DR, and PD-1. Our previous studies demonstrated that while T-cell activation is evidenced by strong upregulation of CD69 and moderate upregulation of CD25 and HLA-DR, it is the increased expression of PD-1 that best reflects the effective PD-1/PD-L1 blockade with antibodies and peptides [6]. Similarly, in the current study, in the presence of PD-L1-blocking antibodies, a significant increase in the expression of PD-1 on the surface of T cells was observed (Fig. 2E and F). This was not observed when PBMCs were cultured with CHO/TCRAct cells that do not overexpress PD-L1, while the contacting of PBMCs with control CHO cells resulted in no activation of T cells. Like in the case of the antibodies, the presence of the peptide pAC65 resulted in a significant increase in the expression of PD-1 on T cells, reflecting the blockade of the PD-1/PD-L1 immune checkpoint (Fig. 2E and F). The presented data give witness to the bioactivity of the peptide pAC65 at sub-nanomolar concentrations and its safety for the tested cells up to micromolar concentrations.

## Discussion

Modulation of immunity has opened up a new stage in the treatment of cancer. To date, all approved immunotherapies targeting the PD-1/PD-L1 interaction use monoclonal antibodies. While non-antibody-based PD-1/PD-L1 inhibitors have many advantages, to date not a single small-molecule inhibitor has been approved for the treatment of cancer and the development of commercially available small-molecule inhibitors remains a challenge. Current non-antibody inhibitors that target PD-L1 fall into three classes: small-molecule inhibitors, linear peptides, and macrocyclic peptides. Among them, the macrocyclic scaffold combines the superior specificity of mAb with a significantly reduced molecular size while providing reduced immunogenicity and increased bioavailability. Compared to the small molecule approaches, the macrocyclic peptides show significantly increased specificity (by 2–3 orders of magnitude) and better binding affinity to PD-L1.

Herein, we described a potent macrocyclic peptide, a PD-L1 inhibitor, that is capable of interfering with both the PD-1/PD-L1 and PD-L1/CD80 complexes formation as verified by AIDA-NMR experiments. However, it does not appear to have any significant affinity for murine PD-L1, which is consistent with our previous study of, for example, the Bristol Myers Squibb macrocyclic peptide p-57 [5]. The structural characterization of the PD-L1/pAC65 complex showed that one molecule of the macrocyclic peptide is bound to a single PD-L1 unit, similarly to other macrocycles and antibodies targeting PD-L1, but unlike small molecules that induce dimerization of this target protein [4, 10]. The comparison of the co-crystal structures of pAC65/PD-L1 with the p57/PD-L1 complex shows the importance of the contribution of polar interactions for the increased potency of the pAC65 macrocycle (Additional file 1: Supplementary materials and methods). The affinity of pAC65 for PD-L1 was verified using a commercially available HTRF assay. The pAC65 peptide dose-dependently dissociated the PD-1/PD-L1 complex with an IC_50_ value of 1.80 ± 0.16 nM, a 40-fold increase in inhibition compared to the activity of the previously published precursor – p57 (IC_50_ 45.4 ± 0.001 nM) [5]. The obtained IC_50_ value suggests that the pAC65 peptide, the structure of which is listed in the Bristol Myers Squib peptide patent [8], may have similar activity to the other known peptide - BMS986189, which entered the clinical trial and provided favorable safety and pharmacokinetic data. The results from a Phase I clinical trial (ClinicalTrials.gov NCT02739373; https://clinicaltrials.gov/ct2/show/NCT02739373) completed in December 2016 led to the development of an analog of the original candidate, BMS986189, for which a new Phase I clinical trial is ongoing in 2022 (ISRCTN Registry ISRCTN17572332, https://www.isrctn.com/ISRCTN17572332). However, according to the information available, the data obtained from this clinical trial will not be disclosed due to its high commercial sensitivity. The peptide pAC65 has also been used as a template for a new generation of macrocyclic peptidomimetics with high biological activity, disclosed by Bristol Myers Squibb in a recent patent application [13].

To test the ability of pAC65 to dissociate the PD-1/PD-L1 complex in the cellular setup, we assessed the effect of pAC65 in an immune checkpoint blockade assay. The pAC65 peptide increased the activation of Jurkat-ECs with an EC_50_ value of 0.58 nM, which was similar to the therapeutic antibody atezolizumab (EC_50_ 0.14 nM), and a spectacular 1000-fold increase in the activation capacity of Jurkat cells compared to p57 (EC_50_ 566 nM, for comparison with other peptides and antibodies see Fig. S9) [5]. The activity of pAC65 was further verified in a T-cell activation assay based on the co-culture of PBMCs and CHO/TCRAct/PD-L1 cells. The presence of the pAC65 peptide induced PD-1 expression on T lymphocytes, causing an effect comparable to that of therapeutic antibodies (atezolizumab, avelumab, and durvalumab).

The co-crystal structure of pAC65 with PD-L1 provides a clear rationale for the observed inhibitory effects of pAC65 on the CD80/PD-L1 and PD-1/PD-L1 interactions. Blocking of both PD-L1/CD80 and PD-1/PD-L1 pathways has also been documented for the other commercial monoclonal antibodies directed against PD-L1: atezolizumab, durvalumab, and avelumab [14]. Also in this respect, the peptide pAC65 presents a clear analogy to the known PD-L1-blocking antibodies.

It has been observed that the binding of CD80 to PD-L1 in a *Cis* mode disallows PD-1 engagement while sparing CD28 co-stimulation [3]. However, the increased PD-L1 levels block T cells *via* the PD-1 co-inhibitor. PD-L1 blockade with antibodies abrogates the latter, but may also diminish the levels of CD80 by promoting its CTLA-4-mediated trans-endocytosis and thus limit the co-stimulation provided by CD28 [3]. In this respect, since the peptide pAC65 is expected to bring similar effects, a combined anti-CTLA-4 treatment seems reasonable for further evaluation of pAC65 activity in vivo.

In another study, the blockade of CD80 with antibodies that disallow the formation of *cis*-PD-L1-CD80 duplexes but do not interfere with CD80 binding to CD28 was shown to liberate PD-L1 and restore proper PD-1 functioning to alleviate autoimmunity [15]. Given the complex nature of the immune checkpoint molecule network, with its properties, the peptide pAC65 not only is a good drug candidate but also a unique non-mAb tool for further investigations.

## Conclusions

In summary, pAC65 is the most potent non-antibody-based PD-1/PD-L1 interaction inhibitor published to date, with the EC_50_ value comparable to the current FDA-approved mAbs, based on our data obtained from multiple bioassays.

The demonstrated bioactivity coupled with the absence of discernible toxicity, as well as the presence of a compound of similar nature – BMS986189 - in several preclinical studies, collectively position pAC65 as an ideal candidate for preclinical testing to verify its potential, safety and tolerability. The discovery of a macrocyclic peptide with mAb equivalent activity could pave the way for the development of macrocyclic peptides that possess pharmacological characteristics that would allow them to be administered by routes such as intranasal, or pulmonary administration, provided that preclinical data are favorable.

### Electronic supplementary material

Below is the link to the electronic supplementary material.

Supplementary Material 1

Supplementary Material 2

Supplementary Material 3

Supplementary Material 4

Supplementary Material 5

Supplementary Material 6

Supplementary Material 7

Supplementary Material 8

Supplementary Material 9

Supplementary Material 10

Supplementary Material 11

## Data Availability

All data generated during this study are included in this published article and its supplementary files. The supplementary data for this article are available at… Crystallographic data are deposited at Protein Data Bank with the accession number 8ALX.

## Abbreviations

APC: antigen-presenting cell
BMS: Bristol Myers Squibb
CD25: the cluster of differentiation 25
CD69: the cluster of differentiation 69
DMSO: dimethyl sulfoxide
EC_50_: half maximal effective concentration
HLA: human leukocyte antigens
*h*PD-1: human PD-1
*h*PD-L1: human PD-L1
IC_50_: half maximal inhibitory concentration
ICB: immune checkpoint blockade
IFN-γ: interferon-gamma
Jurkat-EC: Jurkat effector cells
*m*PD-L1: mouse PD-L1
PBMC: peripheral blood mononuclear cells
PDB: Protein data bank
RMSD: root-mean-square deviation
SD: standard deviation
TCA: T-cell activation
TCRAct: T-cell receptor activator
vIgD: variant Ig domain
