# Supplementary material for: Structural and biological characterization of pAC65, a macrocyclic peptide that blocks PD-L1 with equivalent potency to the FDA-approved antibodies
Source: Mol Cancer. 2023 Sep 7;22:150. doi: 10.1186/s12943-023-01853-4 (PMC10483858; doi:10.1186/s12943-023-01853-4)
Supplement: Supplementary file 1 — Supplementary Material 1 [file 12943_2023_1853_MOESM1_ESM.docx]

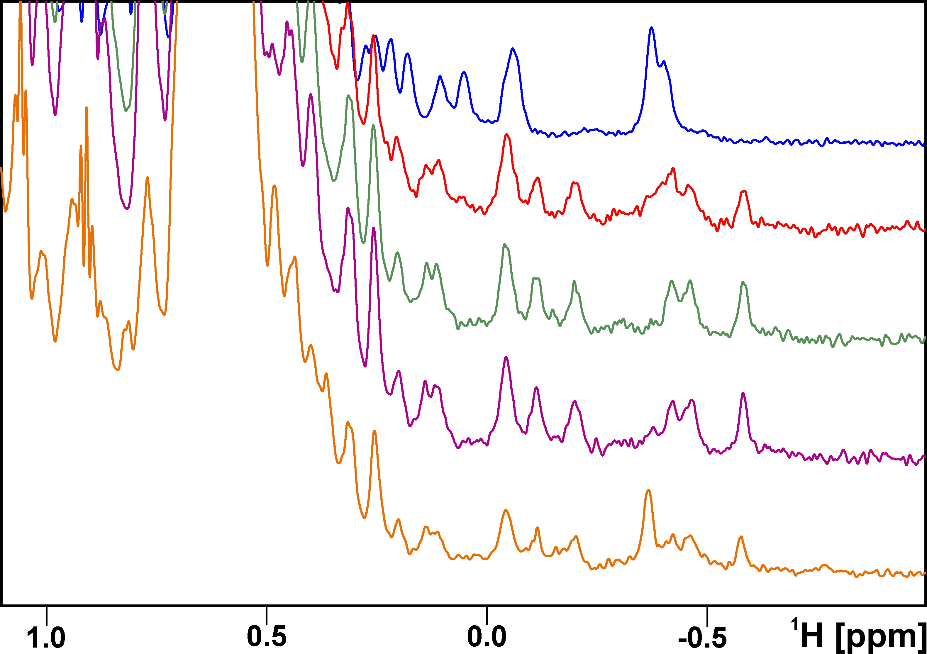


**Figure S1.** ^1^H NMR titration of PD-L1 with peptide pAC65. The aliphatic part of apo-PD-L1 (blue), PD-L1 and pAC65 (red) in molar ratio 10:1, PD-L1 and pAC65 (green) in molar ratio 7:1, PD-L1 and pAC65 (purple) in molar ratio 5:1, and PD-L1 and pAC65 (orange) in molar ratio 1:1.
