# Supplementary figures and images for: Structural and biological characterization of pAC65, a macrocyclic peptide that blocks PD-L1 with equivalent potency to the FDA-approved antibodies
Source: Mol Cancer. 2023 Sep 7;22:150. doi: 10.1186/s12943-023-01853-4 (PMC10483858; doi:10.1186/s12943-023-01853-4)

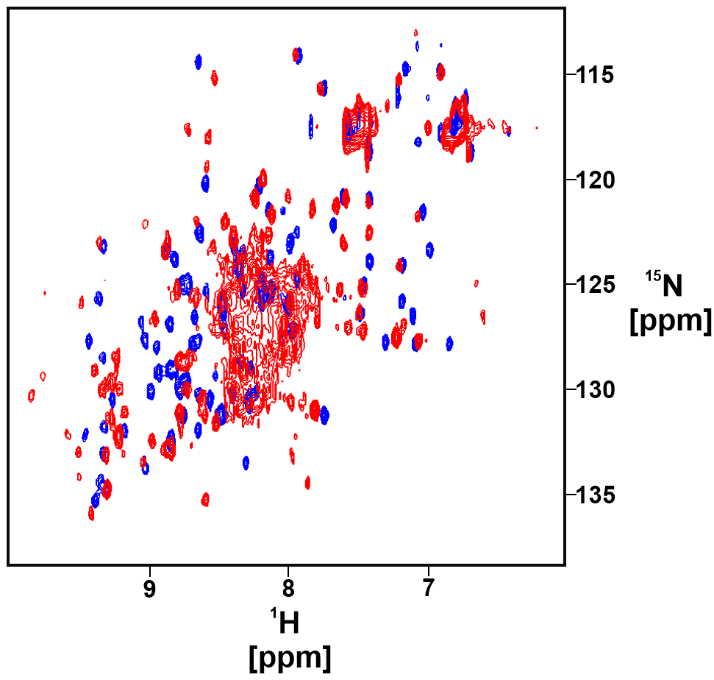


**Figure S2.** 1H-15N SOFASTHMQC spectra of apo-PD-L1 (blue) and peptide pAC65 (red) in the molar ratio 1:1.

Supplement: Supplementary file 2 — Supplementary Material 2 [file 12943_2023_1853_MOESM2_ESM.docx]
