# Supplementary material for: Structural and biological characterization of pAC65, a macrocyclic peptide that blocks PD-L1 with equivalent potency to the FDA-approved antibodies
Source: Mol Cancer. 2023 Sep 7;22:150. doi: 10.1186/s12943-023-01853-4 (PMC10483858; doi:10.1186/s12943-023-01853-4)
Supplement: Supplementary file 3 — Supplementary Material 3 [file 12943_2023_1853_MOESM3_ESM.docx]

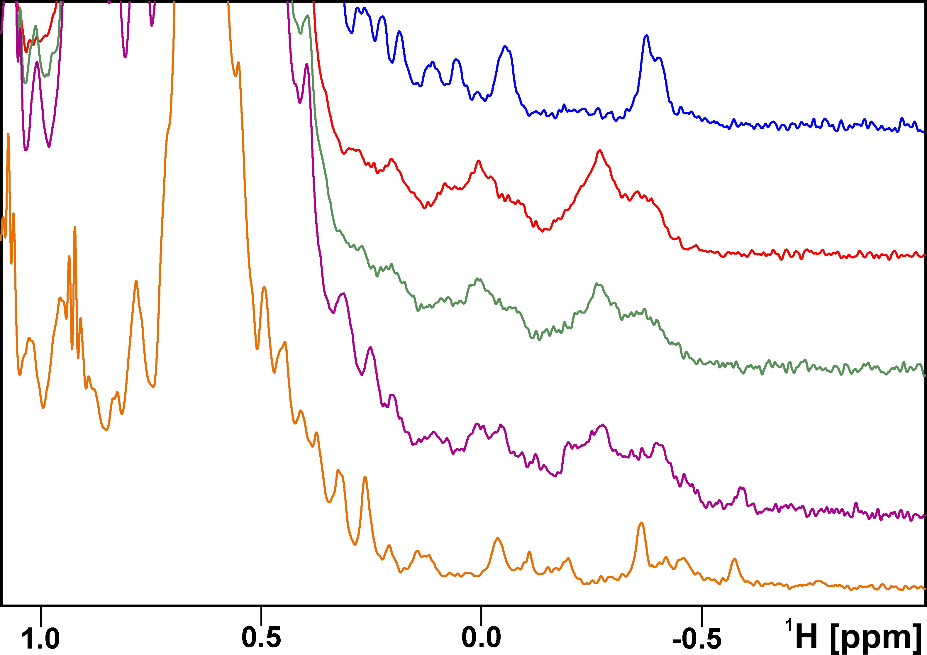


**Figure S4.** The aliphatic part of ^1^H NMR spectra of apo-PD-L1 (blue), apo-CD80 (red), complex of PD-L1/CD80 (green), complex of PD-L1/CD80 and peptide pAC65 in molar ratio 1:1 (purple), and PD-L1 and peptide pAC65 in molar ratio 1:1 (orange).
