# Supplementary material for: Structural and biological characterization of pAC65, a macrocyclic peptide that blocks PD-L1 with equivalent potency to the FDA-approved antibodies
Source: Mol Cancer. 2023 Sep 7;22:150. doi: 10.1186/s12943-023-01853-4 (PMC10483858; doi:10.1186/s12943-023-01853-4)
Supplement: Supplementary file 4 — Supplementary Material 4 [file 12943_2023_1853_MOESM4_ESM.docx]

**Table S1. Data collection and refinement statistics for the PD-L1/pAC65 complex (molecular replacement)**

| **Data collection** | |
| --- | --- |
| Wavelength (Å) | 1.0 |
| Space group | C 2 2 21 |
| Cell dimensions | |
| *a, b, c* (Å) | 56.34 65.47 78.59 |
| *α, β, γ* (o) | 90.00 90.00 90.00 |
| Resolution range (Å) | 42.71 – 1.10 (1.13 - 1.10) |
| *Rmerge* | 0.047 (0.299) |
| *I/σI* | 16.3 (3.7) |
| Completeness (%) | 98.6 (90.50) |
| Redundancy | 5.7 (3.0) |
| Total reflections | 58363 (7707) |
| CC1/2 | 0.999 (0.894) |
| **Refinement statistics** | |
| No. reflections | 58324 (5104) |
| *R*work*/R*free | 0.1190/0.1390 (0.1720)/(0.1820) |
| Wilson B-factor | 8.88 |
| No. atoms | 1474 |
| Protein | 1134 |
| Water | 176 |
| Ramachandran favoured (%) | 97.35 |
| Ramachandran allowed (%) | 2.65 |
| Ramachandran outliers (%) | 0.00 |
| *B*-factors | 13.0 |
| Protein | 10.6 |
| Water | 27.4 |
| R.m.s deviations | |
| Bond lengths (Å) | 0.727 |
| Bond angles (o) | 0.807 |
