# Supplementary material for: Structural and biological characterization of pAC65, a macrocyclic peptide that blocks PD-L1 with equivalent potency to the FDA-approved antibodies
Source: Mol Cancer. 2023 Sep 7;22:150. doi: 10.1186/s12943-023-01853-4 (PMC10483858; doi:10.1186/s12943-023-01853-4)
Supplement: Supplementary file 5 — Supplementary Material 5 [file 12943_2023_1853_MOESM5_ESM.docx]

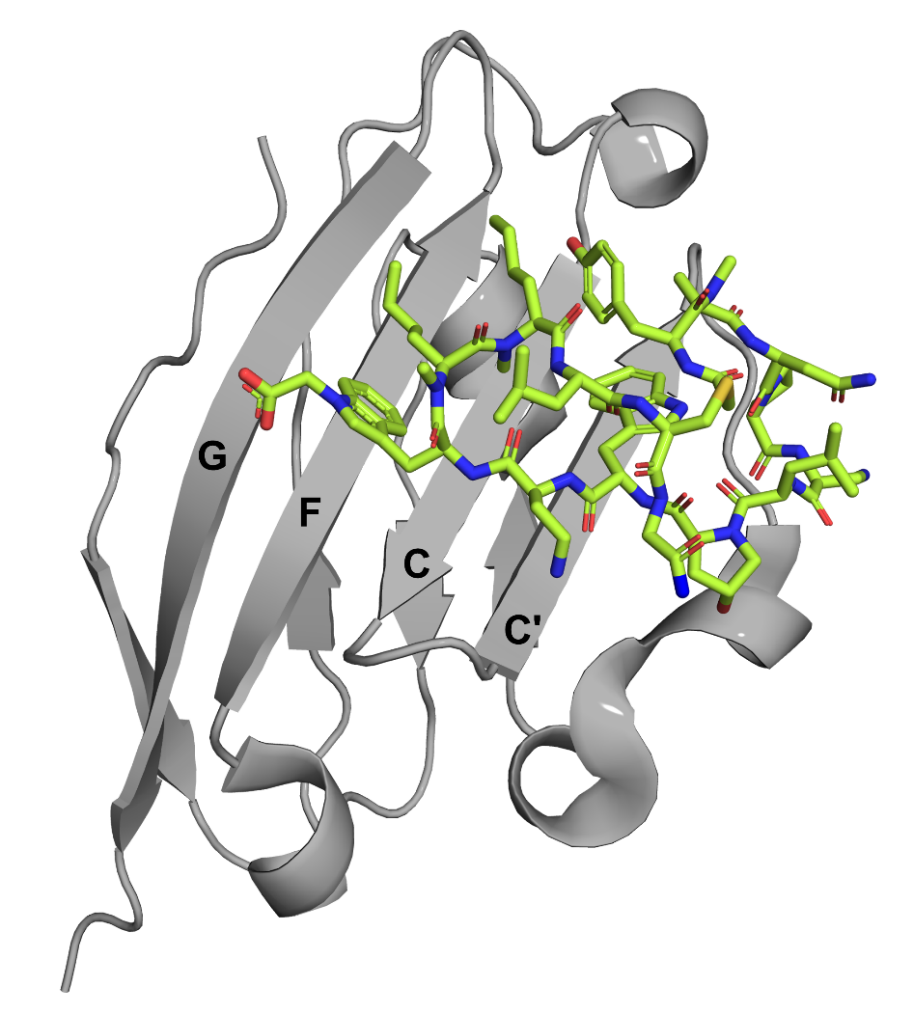


**Figure S6.** Co-crystal X-ray structure of PD-L1/pAC65 complex. pAC65 binds to PD-L1 on the plane of the β-sheet composed by strands G, F, C and C’. PDB: 8ALX.
