# Supplementary material for: Structural and biological characterization of pAC65, a macrocyclic peptide that blocks PD-L1 with equivalent potency to the FDA-approved antibodies
Source: Mol Cancer. 2023 Sep 7;22:150. doi: 10.1186/s12943-023-01853-4 (PMC10483858; doi:10.1186/s12943-023-01853-4)
Supplement: Supplementary file 6 — Supplementary Material 6 [file 12943_2023_1853_MOESM6_ESM.docx]

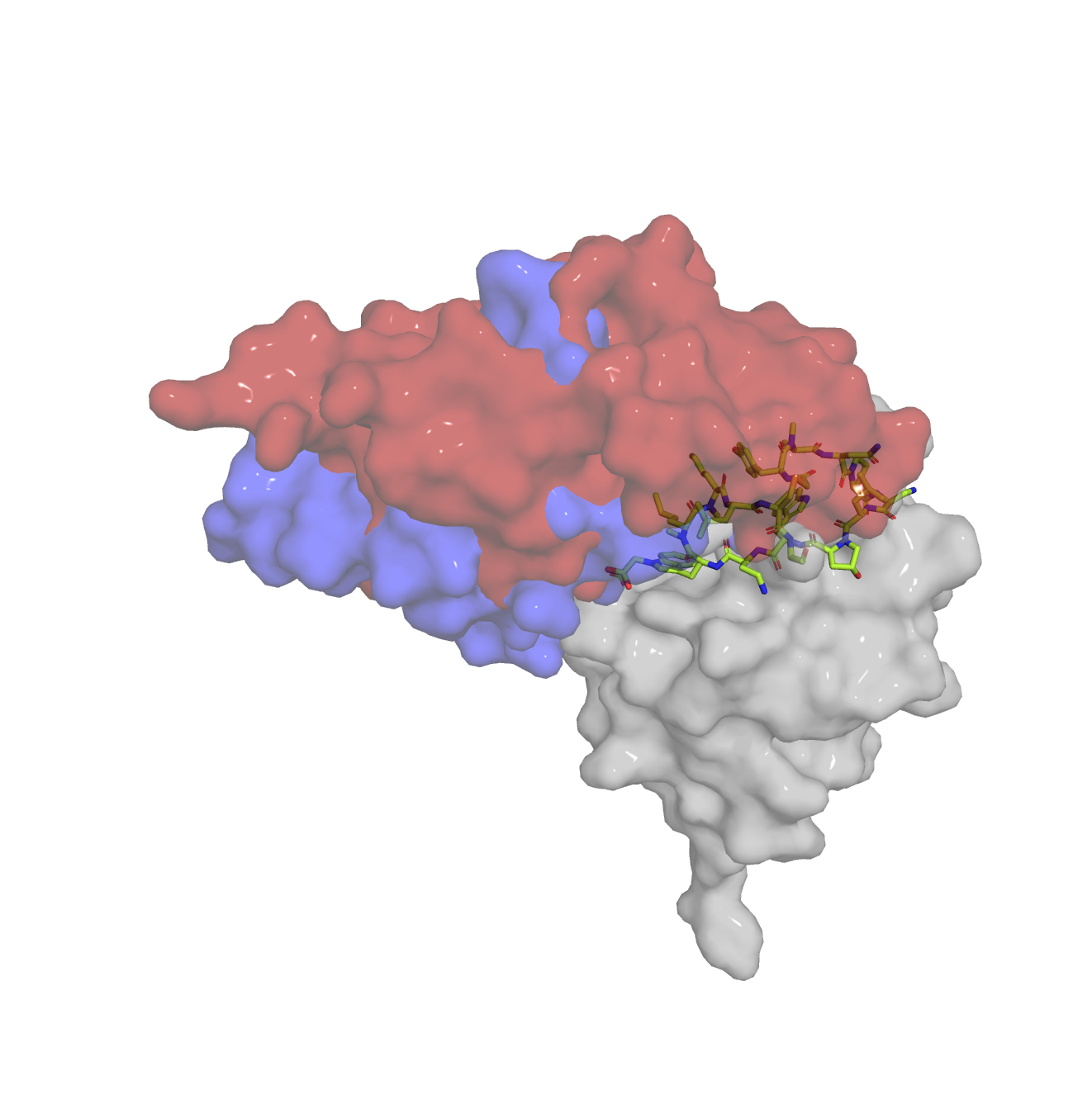


**Figure S7.** Superposition of PD-L1/pAC65 (PDB: 8ALX), PD-L1/PD-1 (PDB: 4ZQK) and PD-L1/ALPN-202 (engineered CD80 vIgD) (PDB: 7TPS) crystal structures. The interaction surface of the pAC65 peptide (green) with PD-L1 (grey) coincides with the binding site of PD-1 (blue) and CD80 mutant (red). The PD-L1 protein from the PD-1/PD-L1 and PD-L1/ALPN-202 complexes is not shown in the figure for clarity.
