# Supplementary material for: Structural and biological characterization of pAC65, a macrocyclic peptide that blocks PD-L1 with equivalent potency to the FDA-approved antibodies
Source: Mol Cancer. 2023 Sep 7;22:150. doi: 10.1186/s12943-023-01853-4 (PMC10483858; doi:10.1186/s12943-023-01853-4)
Supplement: Supplementary file 7 — Supplementary Material 7 [file 12943_2023_1853_MOESM7_ESM.docx]

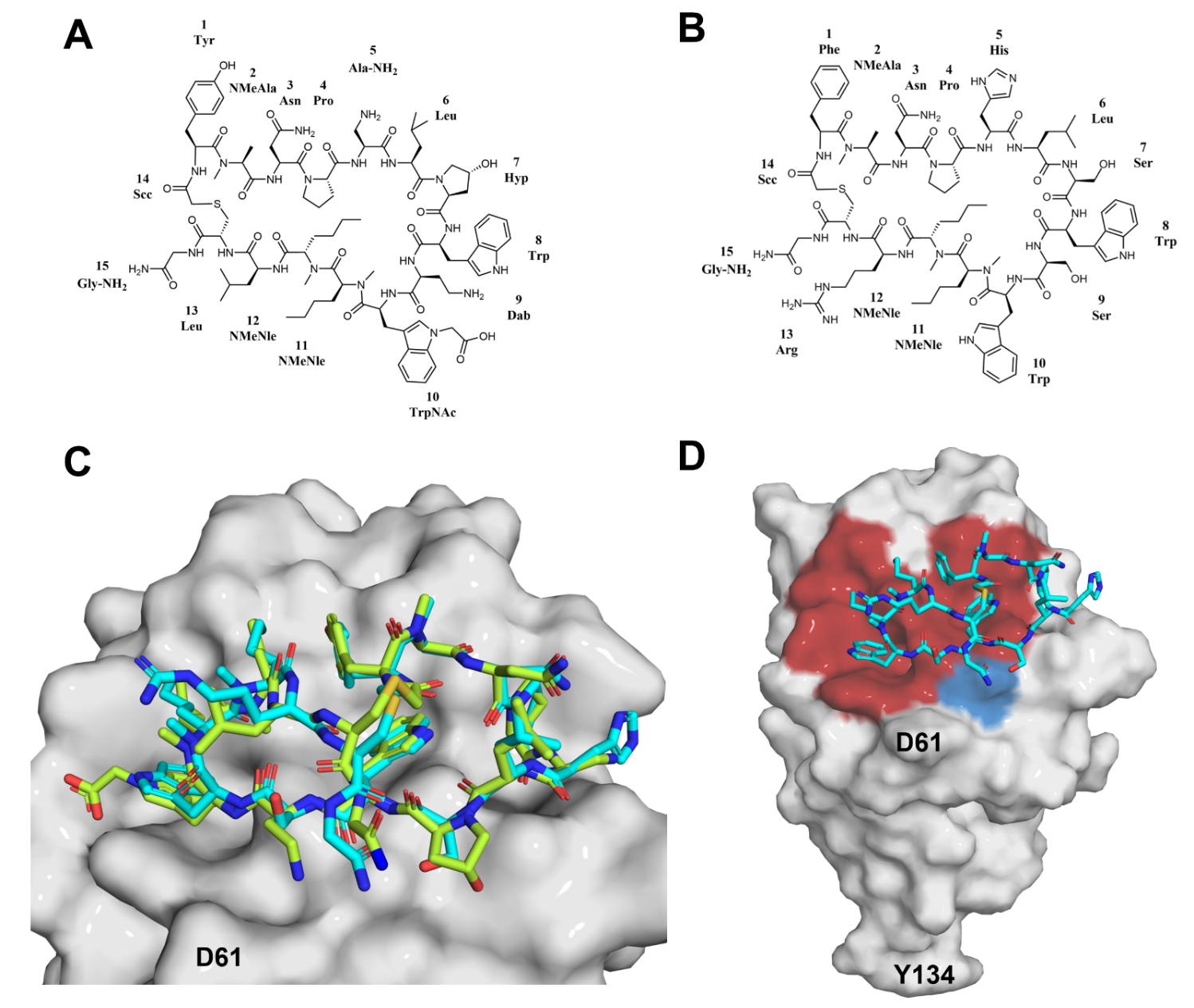


**Figure S8.** Chemical structures and binding mode of macrocyclic peptides at the surface of PD-L1. A) Chemical structure of the pAC65 peptide. B) Chemical structure of the p57 peptide. C) Superposition of the PD-L1/pAC65 (PDB: 8ALX) and PD-L1/p57 (PDB: 5O4Y) complexes (PD-L1 from PD-L1/p57 complex is not shown for clarity). D) PD-L1/p57 (PDB: 5O4Y) binding interface, hydrophobic interactions are shown in red and hydrophilic in blue.
