# Supplementary material for: Structural and biological characterization of pAC65, a macrocyclic peptide that blocks PD-L1 with equivalent potency to the FDA-approved antibodies
Source: Mol Cancer. 2023 Sep 7;22:150. doi: 10.1186/s12943-023-01853-4 (PMC10483858; doi:10.1186/s12943-023-01853-4)
Supplement: Supplementary file 8 — Supplementary Material 8 [file 12943_2023_1853_MOESM8_ESM.docx]

**Supplementary materials and methods**

**Results and discussion**

**Comparison of interactions in PD-L1/pAC65 and PD-L1/p-57 complexes**

The pAC65 peptide contains 15 amino acid residues and belongs to the first group of three independent classes reported by Bristol Myers Squibb (Miller et al., 2016). We previously provided structural characterization of the p-57 peptide which belongs to the same class but shows worse inhibitory activity (Magiera-Mularz et al., 2017). The difference in chemical structures of pAC65 and p-57 peptides concerns 6 of 15 amino acids (Additional file 10: Fig. S8 A and B). The majority of replaced amino acids contain more hydrophilic groups than previous ones directly contributing to the enhanced potency of the inhibitor (Fig. 1 G-I and Additional file 10: Fig. S8 C and D). pAC65 and p-57 peptides utilize the same binding site at the surface of PD-L1 and the main chains of these peptides overlay almost perfectly (root-mean-square deviation [RMSD] of 0.308 Å) (Additional file 10: Fig. S8C). The hydrophobic sidechains bind to the common clefts at the PD-L1 surface providing similar hydrophobic interlinkage in both structures. Although, the differences in the interaction pattern concern primarily polar contacts, the hydrogen bonding network expansion is sufficient for clearly enhanced inhibitory activity of the pAC65 peptide. The enhancement of the interaction via contributing an additional hydrogen bond is particularly prominent for the _65_TrpNAc10 residue. The modification of _57_Trp10 with acetic acid moiety (to _B65_TrpNAc10) caused the extension of initially hydrophobic cleft at the surface of PD-L1 toward two Arg sidechains, which contributes to additional polar forces (the subscript 57 indicates the macrocyclic peptide p-57). The hydroxyl group of _65_Tyr1 residue (replacing _57_Phe1) contributes an additional indirect hydrogen bond mediated by a water molecule. The aromatic rings of _65_Tyr1 and _57_Phe1 overlap perfectly while hydroxyl moiety anchors indirectly at the sidechain of Ser117. Further, the replacement of _57_His5 with _65_Ala-NH_2_5 allowed the position of the amine group in the proximity of side-chain oxygen of Asp73 resulting in the next polar contact formation. Subsequently, the indirect network of hydrogen bonds with Asn63 and Val76 was formed as a result of the introduction of _65_Hyp7 to the pAC65 peptide structure. Replacement of _57_Ser9 with _65_Dab9 resulted again in the formation of two additional polar contacts with the PD-L1 protein (instead of intramolecular interaction contributed by _57_Ser9 in the p-57 molecule). _57_Arg13 is the only polar residue replaced with hydrophobic amino acid - _65_Leu13. However, the Arg sidechain within PD-L1/p-57 complex structure sticks out of the peptide plane to the next protein molecule and is not relevant for peptide-protein interaction.

**Methods**

**The pAC65 peptide synthesis**

All reagents and solvents were purchased from Sigma-Aldrich, CARLO ERBA Reagents, Fluorochem, Novabiochem, and Iris BIOTECH and used without further purification. Fmoc-Trp(CH2COOtBu)-OH was synthesized according to the known protocol (Miller et al., 2016). Peptides were synthesized with an automated microwave solid-phase peptide synthesizer (CEM Liberty Blue) on H-Rink Amide ChemMatrix® resin (loading: 0.59mmol/g) using DMF as a solvent. Coupling procedure was conducted with 0.5 M DIC in DMF (5 eq.) and 0.5 M Oxyma in DMF (5 eq.) and 0.1 M Fmoc-protected amino acids in DMF (5 eq.). Single coupling procedure 4 min at 90°C was used for all amino acids and N-terminal chloroacetic acid with the exception of Cys and amino acids following N-methylated residues. Single coupling was also performed for Cys 10 min at 50°C and amino acids after N-methylated residues were double coupled 2 x 15 min at 75°C and the second coupling was extended for 6h at room temperature. Fmoc deprotection was conducted using 20% piperidine in DMF. Cleavage from resin and deprotection of side chains was performed with the mixture of TFA:tioanisole:EDT:anisole (90:10:6:4) for 3h with shaking. The crude peptide was precipitated with ice-cold diethyl ether and peptide pellet was collected via centrifugation (7000 rpm, 2 x 7 min, 4°C). Peptide was dried with a stream of argon. Cyclization was performed through dissolving crude peptide in the mixture of 0.1 M ammonium bicarbonate buffer pH 8.5 and acetonitrile (2:1, v:v) and stirring for 24h. The solution was evaporated in vacuo. Peptide was purified by HPLC using Knauer Prep with preparative C18 column (Thermo Scientific, Hypersil Gold 12 μm, 250 mm x 20 mm) with water/acetonitrile (0.05% TFA) eluent system. The synthesized peptide was identified by mass spectrometry using WATERS LCT Premier XE system with a time of flight (TOF) and ES ionization. The purity of peptide was confirmed using analytical HPLC Shimadzu with C18 column (Reprosil Sapir 100 C18, 5 μm, 150 x 4.6 mm).

Preparative HPLC: gradient: 0-6 min 20% B; 6-45 min 80% B; 45-55 min 80% B; 55-60 min 20% B, tr = 23.9 min

Analytical HPLC: gradient: 0-2 min 10% B; 2-32 min 90% B; 32-37 min 90% B; 37-40 min 10% B; 40-45 min 10% B, tr = 16.37 min

ESI-MS(+): found 944.4867 [M + 2H+/2], expected 944.486

**Proteins expression and purification**

The PD-L1 protein was obtained according to previously described protocol (Zak et al., 2016). hPD-L1 (amino acids 18-134) was cloned into pET-21b and expressed in *E.coli* BL21 (DE3) at 37 °C overnight; protein expression was induced with 1 mM IPTG and the protein was collected in the form of inclusion bodies, which were subsequently washed and resuspended. Protein was refolded into 0.1 M Tris pH 8.0 containing 1 M L-Arg hydrochloride, 0.25 mM oxidized glutathione and 0.25 mM reduced glutathione by drop-wise dilution manner 50 mg of protein was used per 1 L of refolding buffer. Following the refolding process, three times dialysis was performed against a solution containing 20 mM NaCl and 10 mM Tris-HCl pH 8.0. Finally, the purification of the protein was carried out by size-exclusion chromatography on Superdex 75 (GE Healthcare) in PBS pH 7.4 as elution buffer for NMR and 10 mM Tris pH 8.0 and 20 mM NaCl for crystallization trials, respectively. The folded state and purity of the protein were confirmed by NMR and SDS-PAGE, respectively.

To obtain hCD80, the previously described protocol was used (Ikemizu et al., 2000). Freestyle CHO-S cells (Gibco, R80007) were transfected with hCD80 (extracellular domain, amino acids 1-216) linked to IgG-Fc tag cloned in pcDNA 3.1(+) plasmid. The transfected cells were then selected with G418 antibiotic and single-cell cloning was performed to obtain a cell line stably expressing the hCD80. The protein was purified from cell media (FreeStyle CHO Expression Medium, Gibco, 12651014) using MabSelect SuRe Protein A purification resin (Cytiva, 17543801). The IgG-Fc fragment was cleaved off on the column with Factor Xa Protease (New England Biolabs, P8010L). The final step of the purification was size-exclusion chromatography with 10 mM Tris pH 8.0 and 20 mM NaCl buffer used for NMR measurements. The folded state and purity of the protein were confirmed by NMR and SDS-PAGE, respectively.

**Crystallization of the PD-L1/pAC65 complex**

Purified hPD-L1 was concentrated to 5 mg/ml and mixed with pAC65 in 1:1 molar ratio. Diffraction quality crystals were obtained at room temperature from 1.4 M sodium acetate trihydrate, 0.1 M sodium cacodylate, pH 6.5 using a sitting-drop vapor diffusion setup.

**Structure Determination and Refinement**

Crystals were flash-cooled in liquid nitrogen after cryoprotection. The X-ray diffraction data were collected on the PXIII -X06DA beamline at the Swiss Light Source (Paul Scherrer Institut, Villigen, Switzerland). The data were processed using XDS and scaled using Scala (Evans, 2006; Kabsch, 2010). The initial phases were obtained by molecular replacement with the previous structure of PD-L1 (PDB 5C3T) using Phaser (McCoy et al., 2007; Winn et al., 2011). The model was built using WinCoot and structural refinement was performed with Phenix and PDB-REDO server (Adams et al., 2010; Emsley and Cowtan, 2004; Joosten et al., 2014). The final model was deposited at Protein Data Bank with the accession number 8ALX. The structural models were generated using PyMol (version 2.3.3). The initial X-ray diffraction data were collected at ELETTRA (Trieste, Italy) (Lausi et al., 2015).

**NMR measurements**

Proteins were uniformly ^15^N labelled via expression in minimal medium with ^15^NH_4_Cl as the sole nitrogen source. For NMR measurements, 10% (v/v) of D_2_O was added to the samples to provide lock signal. All spectra were recorded at 300 K using a Bruker Avance 600 MHz spectrometer equipped with a nitrogen-cooled HCN cryogenic probe-head. During the experiment, the ^1^H-^15^N signals were monitored by the SOFAST HMQC (Schanda et al., 2005) (Selective Optimized Flip-Angle Short-Transient Heteronuclear Multiple Quantum Coherence).

**Homogeneous Time-Resolved Fluorescence**

To perform the HTRF assay we used the certified Cis-Bio assay kit at 20 μL final volume using their standard protocol (5 nM of hPD-L1 and 50 nM of hPD-1 in the final formulation). The half maximal inhibitory concentration (IC_50_) of the tested compounds was determined on two individual dilution series. After all components were mixed according to Cis-Bio protocol, the plate was incubated for 2h at room temperature. Next, we performed the TR-FRET measurement on a Tecan Spark 20M. Collected data were background subtracted on the negative control (no anti-PD-1 antibodies), normalized on the positive control (buffer instead of inhibitor solution), averaged, and fitted with a normalized Hill’s equation to determine the IC_50_ value using Mathematica 12.

**Cell culture**

CHO-K1 cells overexpressing T Cell Receptor (TCR) Activator (CHO/TCRAct), CHO-K1 cells overexpressing human PD-L1 (*h*PD-L1), and TCR-Activator (hPD-L1 aAPCs; CHO/TCRAct/PD-L1) and Jurkat T cells (Effector Cells; ECs) overexpressing human PD-1 (*h*PD-1) and a luciferase gene controlled by the NFAT-Response Element were obtained from Promega. These cell lines were cultured in RPMI 1640 medium (Biowest) supplemented with 10% fetal bovine serum (FBS, Biowest) and 200 mM L-glutamine (Biowest). The selection antibiotics: G418 (250 μg/mL, InvivoGen) and Hygromycin B Gold (50 μg/mL, InvivoGen) were also added to the culture medium as per application.

Mouse skin melanoma B16-F10 cell line was purchased from the American Type Culture Collection (ATCC) and cultured in RPMI 1640 medium (Biowest) supplemented with 10% fetal bovine serum (FBS, Biowest) and 200 mM L-glutamine (Biowest). The generation of the B16-F10 cells overexpressing TCR-Activator (*m*aAPCs) clone 3A5 was described in our previous work (Magiera-Mularz et al., 2021). PCR tests for *Mycoplasma* sp. contamination were routinely performed and indicated negative results for both B16-F10 wild-type cells as well as *m*aAPCs clone 3A5 (Van Kuppeveld et al., 1992).

**Immune checkpoint blockade (ICB) assays**

**The *h*PD-1/*h*PD-L1 setup**

The assay was performed according to the manufacturer’s protocol (Promega). CHO/TCRAct/PD-L1 cells were seeded on 96-well white bottom plates at the density of 10,000 cells/well and the next day were co-cultured with Jurkat ECs cells (20,000 cells/well) in the presence of increasing concentrations of the peptide pAC65 in DMSO and DMSO-only as a control (the concentration of DMSO was kept constant at 0.1% (v/v)), and monoclonal antibodies: atezolizumab (Selleckchem), avelumab (MedChemExpress), durvalumab (Selleckchem), MIH5 (Invitrogen), and assay buffer-only (RPMI 1640 + 1% FBS) as a control. The dilutions of monoclonal antibodies were prepared in the assay buffer. Followed by a 6-hour (routinely) or 24-hour (for testing bioactivity in parallel to the *h*PD-1/*m*PD-L1 ICB assay) incubation at 37°C and 5% CO_2_, and an additional 20 min of incubation with the luminescence reagent (Bio-Glo™ Luciferase Assay System, Promega) at room temperature, activation of Jurkat ECs, reflected by luciferase activity, was monitored by luminescence measurements on a Spark microplate reader (Tecan). The data are presented as fold induction of the luminescence signal relative to either DMSO-treated (for pAC65) or untreated (for monoclonal antibodies) cells. Data points represent mean ± SD values from four to eight independent experiments. EC_50_ values as half-maximal effective concentrations were calculated from the Hill curve fitting to the experimental data using Origin Pro 2020 software (OriginLab).

**The *h*PD-1/*m*PD-L1 setup**

B16-F10/TCRAct cells (*m*aAPCs) were seeded on 96-well white bottom plates at the density of 2,000 cells/well and treated with 20 ng/ml of mouse recombinant IFN-γ (Thermo Fisher Scientific) for 48 hours before the ICB assay. Followed by gently washing of culture wells after IFN-γ treatment, Jurkat ECs were added to *m*aAPCs at the density 20,000 cells/well, and cells were co-cultured in the presence of: the peptide pAC65 dissolved in DMSO and DMSO only as a control [constant 0.1% (v/v)], and monoclonal antibodies: atezolizumab (Selleckchem), avelumab (MedChemExpress), durvalumab (Selleckchem), MIH5 (Invitrogen), and assay buffer-only as a control. After 24 hours (37°C, 5% CO_2_) and 20 min of additional incubation with the luminescence reagent (Bio-Glo™ Luciferase Assay System, Promega) at room temperature, activation of Jurkat ECs was monitored by luminescence measurements on a Spark microplate reader (Tecan). Data points represent mean ± SD values from three independent experiments.

**Viability assay**

Jurkat ECs were seeded on 96-well transparent plates in the presence of increasing concentrations of the peptide pAC65 with DMSO-treated cells as a control [constant [0.1% (v/v)]. After 48 hours of incubation, a tetrazolium reagent, Biolog Redox Dye MIX MB (Biolog), was added (6-fold dilution, according to the manufacturer’s instruction), and the culture plates were incubated for an additional 2 hours (37 °C, 5% CO_2_). The absorbance was measured using a Spark microplate reader (Tecan) at 590 nm with 750 nm as a reference. The data are presented as Jurkat ECs survival relative to DMSO-treated cells. Data points represent mean ± SD values from four independent experiments.

**Isolation of PBMCs**

The collection, isolation, and experiments with human Peripheral Blood Mononuclear Cells (PBMCs) were carried out withinethical approvals and collaboration with the Department of Clinical Immunology and Transplantology in Krakow.

Anticoagulant citrate dextrose-A-treated blood from healthy donors was purchased from the Regional Center of Blood Donation and Blood Therapy in Krakow, Poland. PBMCs were isolated from whole blood by density gradient centrifugation using Pancoll human separating solution (PAN-Biotech GmbH). The separated cells were washed and resuspended in RPMI 1640 medium (Biowest) containing 10% FBS (Biowest).

**T-cell activation assay**

CHO-K1, CHO/TCRAct, and CHO TCRAct/PD-L1 were seeded on 24-well transparent plates at the density of 60 000 cells/well. After 24 hours, the PD-L1 antagonists and PBMCs at the density 3,5 x 10^5^ cells per well were added to the culture wells. PD-1/PD-L1 inhibitors that were tested in the T-cell activation assay were: the peptide pAC65 dissolved in DMSO and DMSO-only [0.1% (v/v)] as a control, and monoclonal antibodies: atezolizumab (Selleckchem), avelumab (MedChemExpress), durvalumab (Selleckchem), and assay buffer as a control. The cells were incubated for 48 h (37°C, 5% CO_2_) and then were detached from the plates with TrypLe Select Enzyme (Gibco). PBMCs were stained for 20 min at room temperature with the antibodies: anti-CD4-FITC, anti-CD8-BV510, anti-CD69-APC, anti-CD25-PE, anti-HLA-DR-PerCP, and anti-PD1-PECy7 (Becton Dickinson Biosciences, BD). Following two washing steps, the cells were analyzed using a FACSCanto II cytometer. Data analysis was carried out with FlowJo software, followed by statistical significance calculations done with Origin Pro 2020 software (OriginLab). Statistical significance was analyzed with one-way analysis of variance (ANOVA), followed by the Fisher’s posthoc test: *p < 0.05, **p < 0.01, ***p < 0.01.

**Statistical analysis**

For the analysis of the bioactivity and toxicity of pAC65 in Immune Checkpoint Blockade (ICB) assays, the data points represent mean ± SD values from 3 independent experiments. Statistical analysis was performed using ANOVA with Tukey’s post-hoc test for pairwise comparison with either the untreated control cells: *, p < 0.05, **, p < 0.01, ***, p < 0.001, or DMSO-treated cells: ###, p < 0.001.

For the analysis of the effect of the therapeutic antibodies and the peptide pAC65 on the activation of primary T cells pre-blocked by the PD-L1-overexpressing cells, statistical significance was analyzed with one-way ANOVA, followed by Fisher's posthoc test: * p<0.05, ** p<0.01, ***, p<0.01. Data analysis was carried out with FlowJo software, followed by statistical significance calculations done with Origin Pro 2020 software (OriginLab).

**References**

Adams, P.D., Afonine, P. V., Bunkóczi, G., Chen, V.B., Davis, I.W., Echols, N., Headd, J.J., Hung, L.W., Kapral, G.J., Grosse-Kunstleve, R.W., et al. (2010). PHENIX: a comprehensive Python-based system for macromolecular structure solution. Acta Crystallogr. D. Biol. Crystallogr. *66*, 213–221.

Emsley, P., and Cowtan, K. (2004). Coot: model-building tools for molecular graphics. Acta Crystallogr. D. Biol. Crystallogr. *60*, 2126–2132.

Evans, P. (2006). Scaling and assessment of data quality. Acta Crystallogr. D. Biol. Crystallogr. *62*, 72–82.

Ikemizu, S., Gilbert, R.J.C., Fennelly, J.A., Collins, A. V., Harlos, K., Jones, E.Y., Stuart, D.I., and Davis, S.J. (2000). Structure and dimerization of a soluble form of B7-1. Immunity *12*, 51–60.

Joosten, R.P., Long, F., Murshudov, G.N., and Perrakis, A. (2014). The PDB_REDO server for macromolecular structure model optimization. IUCrJ *1*, 213–220.

Kabsch, W. (2010). XDS. Acta Crystallogr. D. Biol. Crystallogr. *66*, 125–132.

Van Kuppeveld, F.J.M., Van der Logt, J.T.M., Angulo, A.F., Van Zoest, M.J., Quint, W.G.V., Niesters, H.G.M., Galama, J.M.D., and Melchers, W.J.G. (1992). Genus- and species-specific identification of mycoplasmas by 16S rRNA amplification. Appl. Environ. Microbiol. *58*, 2606–2615.

Lausi, A., Polentarutti, M., Onesti, S., Plaisier, J.R., Busetto, E., Bais, G., Barba, L., Cassetta, A., Campi, G., Lamba, D., et al. (2015). Status of the crystallography beamlines at Elettra. Eur. Phys. J. Plus *130*, 43.

Magiera-Mularz K, Skalniak L, Zak KM, Musielak B, Rudzinska-Szostak E, Berlicki Ł, et al. Bioactive Macrocyclic Inhibitors of the PD-1/PD-L1 Immune Checkpoint. Angew Chem Int Ed Engl. 2017;56:13732–5.

Magiera-Mularz, K., Kocik, J., Musielak, B., Plewka, J., Sala, D., Machula, M., Grudnik, P., Hajduk, M., Czepiel, M., Siedlar, M., et al. (2021). Human and mouse PD-L1: similar molecular structure, but different druggability profiles. IScience *24*, 101960.

McCoy, A.J., Grosse-Kunstleve, R.W., Adams, P.D., Winn, M.D., Storoni, L.C., and Read, R.J. (2007). Phaser crystallographic software. J. Appl. Crystallogr. *40*, 658–674.

Miller, M.M., Mapelli, C., Allen, M.P., Bowsher, M.S., Gillis, E.P., Langley, D.R., Mull, E., Poirier, M.A., Sanghvi, N., Sun, L.-Q., et al. (2016). Macrocyclic inhibitors of the PD1/PDL1 and CD80 (B7-1)/PD-L1 protein/protein interactions. (United States patent US 9879046).

Schanda, P., Kupĉe, E., and Brutscher, B. (2005). SOFAST-HMQC experiments for recording two-dimensional deteronuclear correlation spectra of proteins within a few seconds. J. Biomol. NMR *33*, 199–211.

Winn, M.D., Ballard, C.C., Cowtan, K.D., Dodson, E.J., Emsley, P., Evans, P.R., Keegan, R.M., Krissinel, E.B., Leslie, A.G.W., McCoy, A., et al. (2011). Overview of the CCP4 suite and current developments. Acta Crystallogr. D. Biol. Crystallogr. *67*, 235–242.

Zak, K.M., Grudnik, P., Guzik, K., Zieba, B.J., Musielak, B., Dömling, A., Dubin, G., and Holak, T.A. (2016). Structural basis for small molecule targeting of the programmed death ligand 1 (PD-L1). Oncotarget *7*, 30323–30335.
