# Supplementary material for: Structural and biological characterization of pAC65, a macrocyclic peptide that blocks PD-L1 with equivalent potency to the FDA-approved antibodies
Source: Mol Cancer. 2023 Sep 7;22:150. doi: 10.1186/s12943-023-01853-4 (PMC10483858; doi:10.1186/s12943-023-01853-4)
Supplement: Supplementary file 9 — Supplementary Material 9 [file 12943_2023_1853_MOESM9_ESM.docx]

**A**


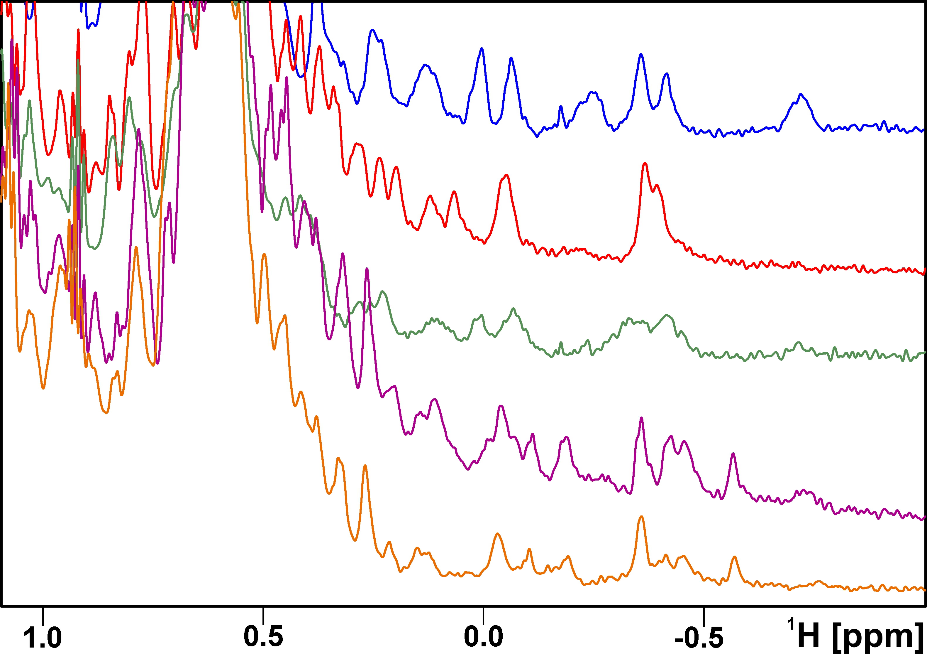


**B**


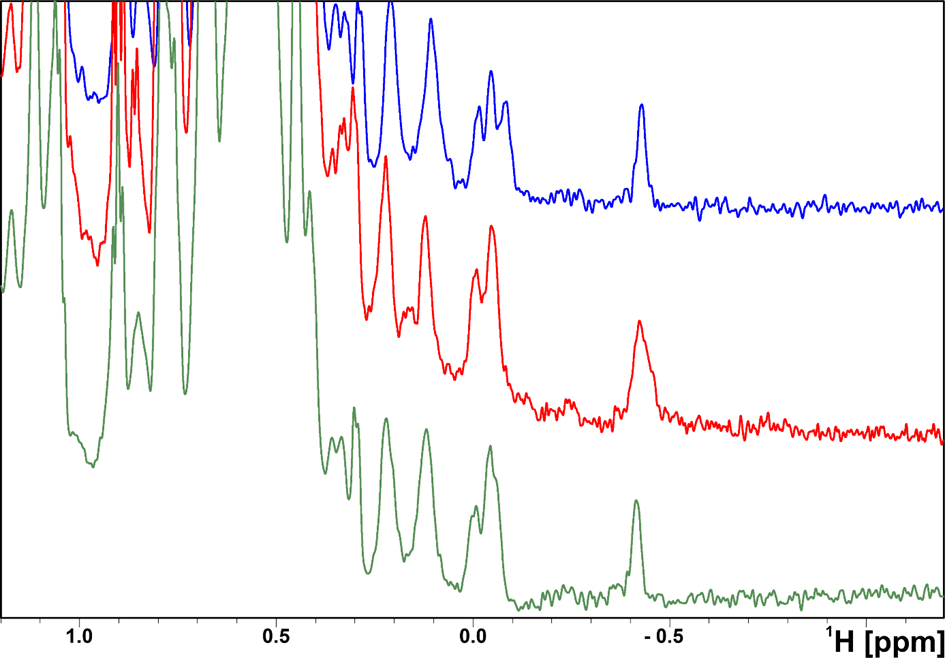


**Figure S3.** **A**: The aliphatic part of ^1^H NMR spectra of apo-PD-1 (blue), apo-PD-L1 (red), complex of PD-1/PD-L1 (green), complex of PD-1/PD-L1 and peptide pAC65 in molar ratio 1:1 (purple), and PD-L1 and peptide pAC65 in molar ratio 1:1 (orange); **B**: Aliphatic part of ^1^H NMR spectra of mPD-L1 (blue), mPD-L1 and pAC65 peptide in molar ratio 1:2 (red), respectively, and mPD-L1 and DMSO-d6 as a blank (without inhibitor) (green).
