# Supplementary material for: Structural and biological characterization of pAC65, a macrocyclic peptide that blocks PD-L1 with equivalent potency to the FDA-approved antibodies
Source: Mol Cancer. 2023 Sep 7;22:150. doi: 10.1186/s12943-023-01853-4 (PMC10483858; doi:10.1186/s12943-023-01853-4)
Supplement: Supplementary file 10 — Supplementary Material 10 [file 12943_2023_1853_MOESM10_ESM.docx]

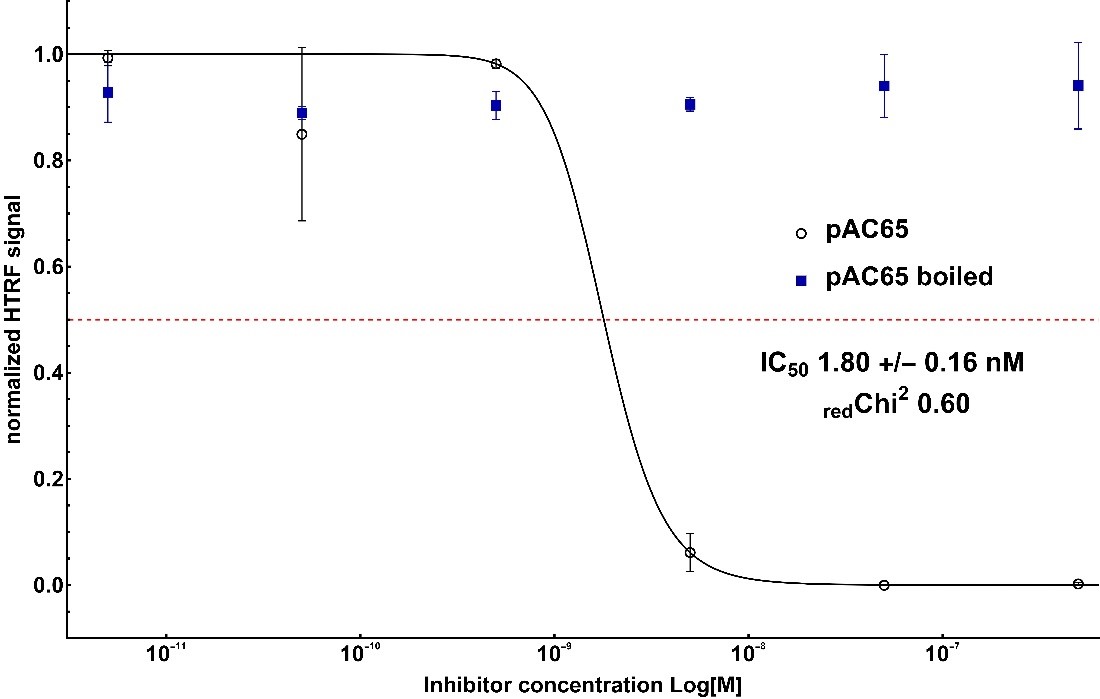


**Figure S5.** HTRF data for the PD-L1/PD-1 complex dissociation assay with the peptide pAC65 (circles). Data points are the average values from 2 independent experiments. The fit of simplified Hill’s equation is shown in black. In blue squares, a negative control – boiled pAC65.
