# Supplementary material for: Structural and biological characterization of pAC65, a macrocyclic peptide that blocks PD-L1 with equivalent potency to the FDA-approved antibodies
Source: Mol Cancer. 2023 Sep 7;22:150. doi: 10.1186/s12943-023-01853-4 (PMC10483858; doi:10.1186/s12943-023-01853-4)
Supplement: Supplementary file 11 — Supplementary Material 11 [file 12943_2023_1853_MOESM11_ESM.docx]

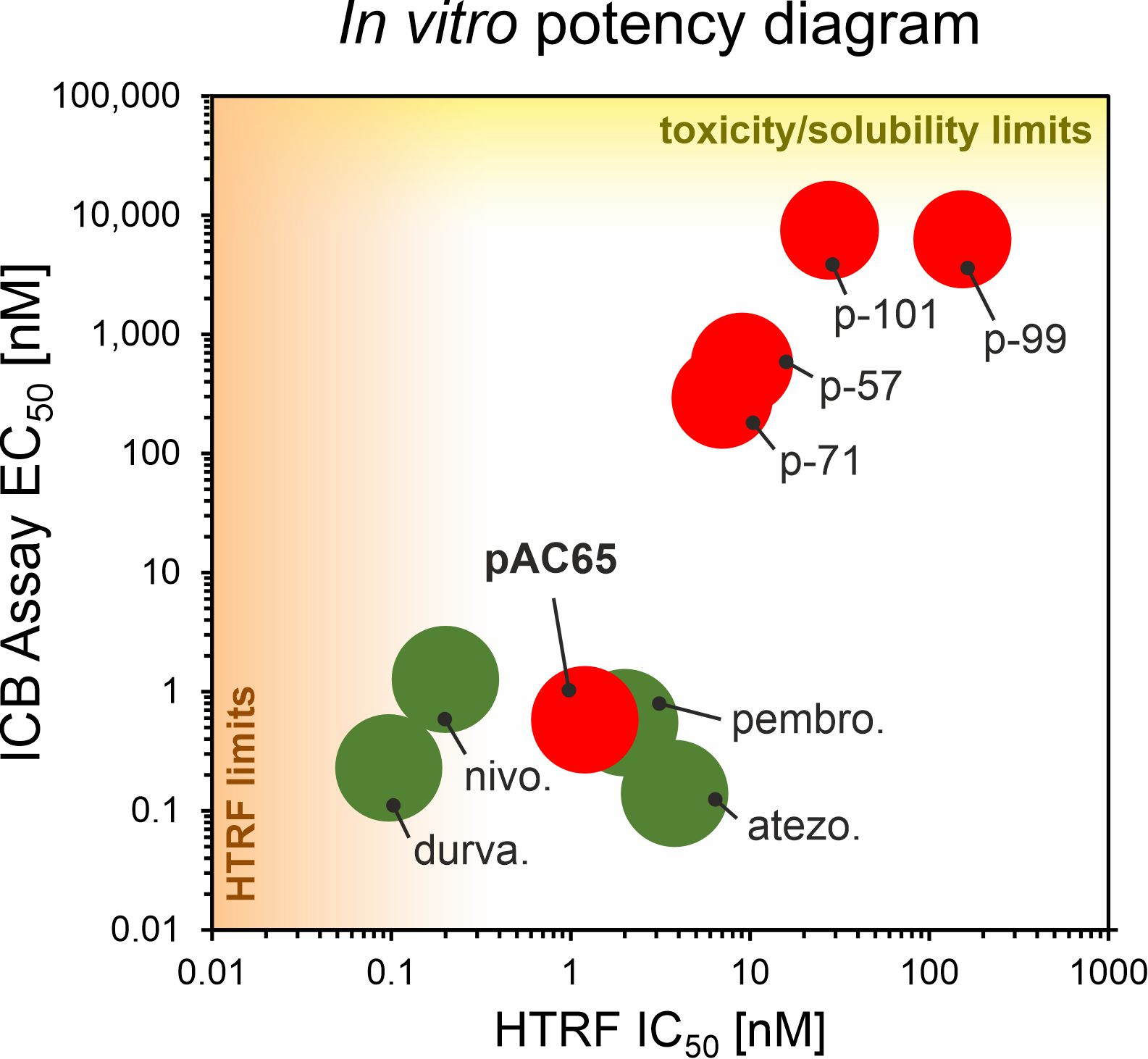


**Figure S9.** Correlation of IC_50_ values derived from the HTRF assay and EC_50_ values derived from the ICB assay for representative macrocyclic peptides (red) and therapeutic antibodies (green). “HTRF limit” indicates a bottom limit of IC_50_ determination with HTRF (based on the concentrations of targeted PD-1 and PD-L1 proteins). “Toxicity/solubility limits” indicate the upper limits of EC_50_ determination in the ICB assay (related to cell toxicity and limited water solubility of the molecules). Atezo., atezolizumab; durva., durvalumab; nivo., nivolumab; pembro., pembrolizumab. Graph adapted from *Surmiak et al.*, 2021, DOI: 10.3390/ijms222111797.
